# Role of HOX Genes in Stem Cell Differentiation and Cancer

**DOI:** 10.1155/2018/3569493

**Published:** 2018-07-22

**Authors:** Seema Bhatlekar, Jeremy Z. Fields, Bruce M. Boman

**Affiliations:** ^1^Center for Translational Cancer Research, Helen F. Graham Cancer Center and Research Institute, Newark, DE, USA; ^2^Department of Biological Sciences, University of Delaware, Newark, DE, USA; ^3^CATX Inc., Princeton, NJ, USA; ^4^Kimmel Cancer Center, Thomas Jefferson University, Philadelphia, PA, USA

## Abstract

HOX genes encode an evolutionarily conserved set of transcription factors that control how the phenotype of an organism becomes organized during development based on its genetic makeup. For example, in bilaterian-type animals, HOX genes are organized in gene clusters that encode anatomic segment identity, that is, whether the embryo will form with bilateral symmetry with a head (anterior), tail (posterior), back (dorsal), and belly (ventral). Although HOX genes are known to regulate stem cell (SC) differentiation and HOX genes are dysregulated in cancer, the mechanisms by which dysregulation of HOX genes in SCs causes cancer development is not fully understood. Therefore, the purpose of this manuscript was (i) to review the role of HOX genes in SC differentiation, particularly in embryonic, adult tissue-specific, and induced pluripotent SC, and (ii) to investigate how dysregulated HOX genes in SCs are responsible for the development of colorectal cancer (CRC) and acute myeloid leukemia (AML). We analyzed HOX gene expression in CRC and AML using information from The Cancer Genome Atlas study. Finally, we reviewed the literature on HOX genes and related therapeutics that might help us understand ways to develop SC-specific therapies that target aberrant HOX gene expression that contributes to cancer development.

## 1. HOX Genes

**Figure 1:**
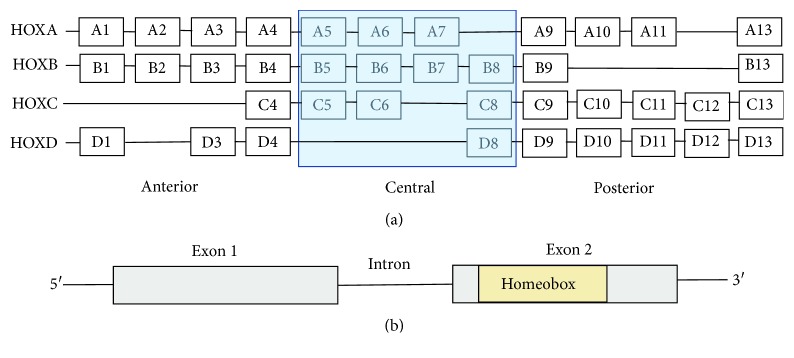
HOX genes and genome organization. (a) In humans, there are a total of 39, clustered into four families, namely, HOXA, HOXB, HOXC, and HOXD. Each family consists of 13 paralogous groups with nine to eleven numbers assigned based on their sequence similarity and position within the cluster. (b) HOX genes have two exons and 1 intron. Exon 2 has a 120-nucleotide sequence, called a Homeobox that encodes a 61 amino acid HOX protein.

*HOX* genes are master transcriptional regulators that have diverse roles from embryogenesis to carcinogenesis. The HOX genes are an evolutionary conserved family of genes that control anterior-posterior axis and dorsal-ventral anatomic development during embryogenesis. In humans there are a total of 39 HOX genes situated in clusters on four different chromosomes (7p15, 17q21.2, 12q13, and 2q31). These clusters are named as four HOX families: HOXA, HOXB, HOXC, and HOXD. Each family consists of 13 paralog groups with nine to eleven numbers assigned based on their sequence similarity and position within the cluster (Figure 1(a)). HOX genes contain two exons and a single intron. Exon 2 contains a 120-nucleotide sequence, known as homeobox. This homeobox encodes a 61 amino acid helix-turn-helix motif known as a homeodomain (Figure 1(b)). The protein products of the HOX genes are transcription factors that are capable of binding to specific nucleotide sequences on the DNA.

### 1.1. HOX Genes and Stem Cell Differentiation

SCs are multipotent cells that have the ability to self-renew or to differentiate along multiple lineages. HOX genes have been shown to play crucial roles during SC differentiation from embryonic stages of development to tissue-specific SC functions. Of the 39 HOX genes, mutations in 10 HOX genes (*HOXA1*, *HOXA2*, *HOXA11*, *HOXA13*, *HOXB1*, *HOXB13*, *HOXC13*, *HOXD4*, *HOXD10*, and *HOXD13*) have been found to cause human disorders with variations in their inheritance patterns, penetrance, expressivity, and mechanisms of pathogenesis [1]. Congenital defects caused by mutations in HOX genes support the concept that HOX gene function is crucial for SCs during development and differentiation. Therefore, we reviewed the published literature for the role of HOX genes during differentiation of three main types of SC, namely, (i) embryonic SCs, (ii) adult SCs (hematopoietic SCs, colonic SCs, and mesenchymal SCs), and (iii) induced pluripotent SCs.

#### 1.1.1. HOX Genes and Embryonic Stem Cell Differentiation

Embryonic stem cells (ESCs) are obtained from the inner cell mass of the blastocyst. ESCs are pluripotent cells that can give rise to most cell types except the placenta and umbilical cord. Retinoic acid (RA) signaling regulates HOX gene expression in ESCs during embryonic development. In adult neurogenesis, ESCs treated with RA almost exclusively differentiate into neurons and develop a HOX-related expression profile [2]. Retinoic acid response elements (RAREs) are found in regulatory regions of many HOX genes [3, 4]. In mice, RAREs are found in *Hoxa1*, *Hoxa4*, *Hoxb1*, *Hoxb4*, and *Hoxd4* [5–14]. Several HOX genes have been found to be strongly upregulated during differentiation in the presence of RA [15]. RA receptor *γ* (RAR*γ*) was found to be essential for RA-induced HOX gene transcriptional activation in ESCs. Deletion of its binding site in the *Hoxa1* enhancer attenuates transcriptional and epigenomic activation of both Hoxa and Hoxb gene clusters. It was reported that RA/RAR*γ* signaling is critical for the removal of histone methylation occurring on the amino terminal tail core of the core histone H3 (H3K27me3) from activated Hox genes during ESC differentiation [16]. The entire Hox cluster is actively repressed in ESCs by polycomb repressor complexes and plays key regulatory roles during their differentiation to multipotent progenitors in developing tissues [17]. As ESCs differentiate into different lineages, Hox gene clusters become activated in a controlled and sequence-specific manner [17, 18]. It has been shown that timely induction of *Hoxb1* in ESCs results in the differentiation of neuronal SCs and neural progenitors of distinct posterior identities [19]. It is well-known that *Hoxb4* overexpression in ESCs confers long-term repopulating ability to ESC-derived hematopoietic stem cells (HSCs). Furthermore, *Hoxb4* acts as a master regulator of ESC differentiation into HSCs by directly targeting multiple essential hematopoietic transcription factors and epigenetic factors [20]. Overall, these results show that complex regulatory mechanisms exist through which Hox genes are expressed in ESCs and function during differentiation.

#### 1.1.2. HOX Genes and Adult Stem Cells

Adult SCs, also referred to as somatic or tissue-specific SCs, give rise to different cell types that are specific to each tissue type or organ in which they reside. HOX genes are crucial for the maintenance and functioning of adult SCs. Here, we focus on three types of adult SCs: hematopoietic SCs that generate entire blood cell lineages, colonic SCs that reside at the base of the normal crypt and are responsible for colonic tissue renewal and proper functioning of the colon, and mesenchymal SCs isolated from the stroma and that generate various differentiated cell types from bone, cartilage, and fat cells.

**Figure 2:**
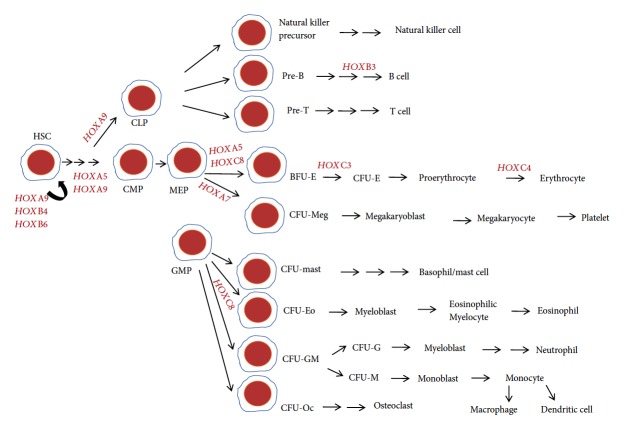
HOX gene expression during hematopoiesis. The hematopoietic stem cell (HSC) is a multipotent stem cell that has the ability to give rise to common lymphoid progenitor (CLP) and common myeloid progenitor (CMP) cells. *HOXA9*, *HOXB4*, and *HOXB6* are known to be expressed in HSC and regulate HSC self-renewal. *HOXA5* and *HOXA9* are involved in the proliferation and differentiation of HSC to CMP, and *HOXA9* regulates the differentiation of HSC into CLP. *HOXB3* is expressed during the differentiation of pre-B cells into B cells. *HOXA5* and *HOXC8* are expressed during erythroid differentiation of megakaryocyte-erythrocyte progenitors (MEP) whereas *HOXA7* is expressed during megakaryocyte differentiation. *HOXC3* and *HOXC4* are crucial during erythroid lineage differentiation. *HOXC8* is shown to play a regulatory role during the differentiation of granulocyte-monocyte progenitor (GMP) cells. HSC: hematopoietic stem cells; CMP: common myeloid progenitor; CLP: common lymphoid progenitor; MEP: megakaryocyte-erythrocyte progenitor; GMP: granulocyte-monocyte progenitor; BFU-E: erythroid burst-forming units; CFU-E: erythroid colony-forming unit; CFU-Meg: megakaryocyte colony-forming unit; CFU-mast: mast colony-forming unit; CFU-Eo: eosinophil colony-forming unit; CFU-GM: granulocyte-monocyte colony-forming unit; CFU-Oc: osteoclasts colony-forming unit.

*(1) HOX Genes and Hematopoietic Stem Cells*. Hematopoietic stem cells (HSCs) are generally found in the bone marrow but can also be isolated from the peripheral blood, umbilical cord blood, and fetal liver. HOX genes are expressed in HSCs and progenitors in a lineage-specific and differentiation stage-restricted manner. For example, *HOXB3*, *HOXB4*, and *HOXA9* are abundantly expressed in HSCs, whereas *HOXB8* and *HOXA10* are expressed only in myeloid committed cells. Recent studies showed that HOXA family genes are mostly expressed in the myeloid cells, HOXB family genes are mostly expressed in the erythroid cells, and HOXC family genes are commonly seen in the lymphoid cells. HOXD family genes are not expressed during hematopoiesis despite their similarities to other HOX gene clusters [21–24]. Since specific HOX genes are essential for SC differentiation into specific blood cell types, we present a diagram of all known HOX genes associated with hematopoiesis (Figure 2).

#### 1.1.3. *HOXA5*

*HOXA5* overexpression in bone marrow CD34+ SCs and cord blood CD34+ CD38− cells resulted in a significant shift in myeloid differentiation [25]. When *HOXA5* was overexpressed in HSCs, erythroid progenitors (burst-forming unit-erythroid BFU-E) were significantly decreased in frequency among all progenitors, with no reduction in total colony-forming unit (CFU) numbers [25]. Similarly, the overexpression of *HOXA5* inhibited erythroid differentiation of K562 cells [26]. In contrast, the knockdown of *HOXA5* in human bone marrow cells resulted in the inhibition of granulocytic/monocytic hematopoiesis and increased erythroid progenitors [26]. Overall, these studies showed that *HOXA5* is crucial for the balance between myeloid and erythroid differentiation.

#### 1.1.4. *HOXA7*

*Hoxa7* knockout mice showed reduced megakaryocytic/erythroid progenitors (MEP) and exhibited reticulocytosis and thrombocytopenia without anemia [27], suggesting that *HOXA7* is required for MEP differentiation.

#### 1.1.5. *HOXA9*

*HOXA9* is one of the most abundant HOX genes in HSCs. *HOXA9* is downregulated during HSC differentiation [24, 28]. *Hoxa9* knockout mice displayed marked lymphopenia (low levels of lymphocytes in the blood) and substantial reductions of common lymphoid progenitors (CLPs) and lymphoid precursors. In addition, significant reduction of common myeloid progenitors (CMPs) and granulocyte/monocyte progenitors (GMPs) was observed upon *Hoxa9* knockout *in vivo* [27]. *Hoxa9* knockout mice also showed a reduction in the number of total leukocytes [29], and the loss of expression of *Hoxa9* impaired the proliferating and repopulating ability of HSCs [30]. *HOXA9* thus appears to regulate HSC proliferation, self-renewal, and myeloid and lymphoid differentiation.

#### 1.1.6. *HOXA10*

*HOXA10* overexpression in human cord blood and fetal liver CD34+ cells resulted in a significant production of blast cells (undifferentiated blood cells, commonly seen in acute leukemia) and myelopoiesis concomitant with a complete block of erythroid differentiation and a severe reduction in B cell development [31]. Thus, these findings suggest that the regulation of *HOXA10* expression is crucial for preventing abnormal development and differentiation of HSCs.

#### 1.1.7. *HOXB3*

In mice, the overexpression of *Hoxb3* blocked B and T cell differentiation and caused a delay in myeloid precursor proliferation [32], whereas the knockout of *Hoxb3* in mice at 6 months of age caused significant impairment of B cell development in the bone marrow [33].

#### 1.1.8. *HOXB4*

*HOXB4* is known to enhance primitive hematopoietic cell growth by increasing self-renewal without affecting homeostatic control of HSC population size or of the rate of HSC production. The retention of full lymphomyeloid repopulating potential and enhanced *in vivo* regenerative potential demonstrates the feasibility of achieving significant ex vivo expansion of HSCs without functional impairment [34–39]. *Hoxb3* and *Hoxb4* double-knockout mice showed defects in endogenous hematopoiesis with reduced cellularity of HSC regeneration. *Hoxb3^−^/Hoxb4^−^* mice showed reduction in hematopoietic progenitor numbers without perturbing lineage commitment [40].

#### 1.1.9. *HOXB6*

*Hoxb6* overexpression in mice resulted in HSC and myeloid progenitor cell expansion but inhibited erythropoiesis and lymphopoiesis [41]. Upregulation of *HOXB6* is often seen in acute myeloid leukemia (AML). Cytogenetic analysis of a subset of *HOXB6*-induced AMLs revealed recurrent deletions of chromosome band 2D-E4, a region frequently deleted in *HOXA9*-induced AMLs. The biologic effects of *HOXB6* were seen to be largely dependent on DNA binding but they were independent of direct interaction of *PBX1* [41]. The knockout of *Hoxb6* resulted in an increase in early erythroid progenitors in murine bone marrow and fetal liver cells [42]. Thus, *HOXB6* is critical not only for HSC self-renewal and maintenance but also for regulatory balance between lymphoid and myeloid differentiation.

#### 1.1.10. *HOXC3*

An antisense oligonucleotide against *Hoxc3* inhibited the formation of colony-forming units (CFUs) of erythroid-derived colonies without any changes in size or degree of hemoglobinization. Early erythroid burst-forming unit colonies or myeloid colonies were not affected, demonstrating that *Hoxc3* is involved in an early step in proliferation of the erythroid colony-forming unit subset of progenitor cells [43].

#### 1.1.11. *HOXC4*

The enforced expression of *HOXC4* in human CD34+ cells induced a significant increase in the number of erythroid colonies compared with granulocyte/macrophage colony-forming units (CFU-GM), without perturbing, at least *in vitro*, the maturation program of these cells. On the other hand, *HOXC4* overexpression did not induce any skewing in the colony types derived from the myeloid lineage [44].

#### 1.1.12. *HOXC8*

A significant reduction in the number of erythroid burst-forming units (BFU-E) and in CFU-GM occurred in *Hoxc8* null mice, although the peripheral blood cell counts were normal [45] suggesting that *HOXC8* plays a role during MEP (megakaryocyte-erythroid progenitor) differentiation into BFU-E and GMP differentiation into CFU-GM.

**Figure 3:**
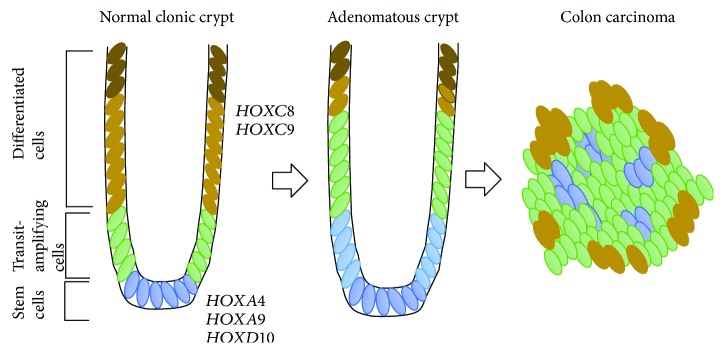
HOX gene expression during colonocyte differentiation. Normal colonic crypts consist of mainly three types of cells based on their location in the crypt. Colon stem cells (SCs) reside at the base of the colonic crypt (shown in blue color). *HOXA4*, *HOXA9*, and *HOXD10* are expressed in colonic SCs and regulate colonic crypt SC differentiation [49, 50]. SCs generate transit-amplifying cells (shown in green color) that are actively proliferating and differentiating (shown in gold-bronze yellow color) as they move up the axis in the colonic crypt. Finally, fully differentiated or terminally differentiating cells are found at the top of the crypt (shown in brown color). Studies have shown that HOXA family genes are expressed mostly in proliferating colonic cells, and HOXC family genes are expressed in differentiating cells [68]. HOXB and HOXD family genes are expressed throughout the colonic crypts [68]. In colon tumors, the dysregulation of *HOXA4* and *HOXA9* in colon SCs caused aberrant self-renewal and proliferation, contributing to colon carcinoma [50]. *HOXC8* and *HOXC9* are expressed in the differentiating cells in the colonic crypt [68].

*(1) HOX Genes and Colonic Stem Cells*. We previously showed that normal colonic SCs are found at the base of the normal human colon crypt and can be isolated using SC markers such as aldehyde dehydrogenase (*ALDH1*), *ALCAM* (CD166), and *LGR5* [46–48]. The overpopulation of colonic SCs drives colorectal cancer (CRC) development [47, 48]. We studied the expression of HOX genes in normal colonic SCs by microarray profiling. Our analysis showed that *HOXA4*, *HOXA9*, and *HOXD10* are expressed more in colonic SCs than in proliferating cells or differentiating crypt cells [49]. Further studies showed that *HOXA4* and *HOXA9* are enriched in SCs during CRC development and that the dysregulation of *HOXA4* and *HOXA9* expression promotes self-renewal and proliferation of colonic SCs [50] (Figure 3). The siRNA knockdown of *HOXA4* and *HOXA9* in colon cancer cell lines SW480 and HT29 reduced proliferation and sphere-forming ability of colon SCs [50] thus suggesting regulatory roles of HOX genes during colon SC maintenance and differentiation.

*(2) HOX Genes and Mesenchymal Stem Cells*. Mesenchymal stem cells (MSCs) isolated from the umbilical cord blood express *HOXA9*, *HOXB7*, *HOXC10*, and *HOXD6*, whereas bone marrow-derived MSCs express *HOXB7* and *HOXD6* [51]. *HOXC10* was found to be differentially expressed in amnion- and decidua-derived MSCs [51]. HOX genes, particularly *HOXA9*, *HOXB7*, *HOXC10*, and *HOXD8*, were used as biomarkers to distinguish between MSCs derived from unrestricted somatic stem cells and cord blood [51]. A study by Woo et al. [52] showed that the expression of *HOXC13* increased whereas *HOXD13* expression decreased as bone marrow-derived MSCs differentiated into osteoblasts during osteogenesis. Taken together, these findings indicate that distinct expression patterns of *HOXA5*, *HOXA10*, *HOXB6*, *HOXB7*, *HOXC4*, *HOXC6*, *HOXC8*, *HOXC9*, *HOXC10*, *HOXD3*, and *HOXD8* exist in MSCs derived from different human sources [52]. *Hoxb2*, *Hoxb5*, *Hoxb7*, and *Hoxc4* genes were found to regulate self-renewal and differentiation of murine MSCs [53].

HOX genes have been shown to play critical roles during osteogenesis of human MSCs. Histone demethylase KDM6B controlled HOX expression by removing histone 3K27 trimethylation (H3K27me3) and reduced KDM6B significantly by reducing osteogenic differentiation and increasing adipogenic differentiation [54]. The role of HOX genes during differentiation of human vascular wall-resident CD44+ multipotent stem cells (VW-MPSCs) was also studied [55]. VW-MPSCs are capable of differentiating into pericytes and smooth muscle cells. This study demonstrated that the expression of *HOXB7*, *HOXC6*, and *HOXC8* is differentially expressed in VW-MPSCs as compared to terminally differentiated human aortic smooth muscle cells, endothelial cells, and undifferentiated pluripotent ESCs. The knockdown of HOX genes in VW-MPSCs reduced their sprouting capacity and increased their levels of smooth muscle markers, transgelin and calponin, as well as histone H1. In addition, changes in methylation patterns of the TAGLN promoter were observed [55]. Overall, this study suggested a role for HOX genes in regulating differentiation of human VW-MPSC into smooth muscle cells via epigenetic mechanisms. The results of this study will help us understand VW-MPSC-dependent vascular disease processes such as neointima formation and tumor vascularization [55].

#### 1.1.13. HOX Genes and Induced Pluripotent Stem Cells

Induced pluripotent stem cells (iPSCs) are cells that are engineered in the lab by converting tissue-specific adult SCs into cells that possess ESC-like properties. iPSCs, like ESCs, did not express HOX genes [56]. Although suppression of HOX gene expression was observed in iPSCs, transient WNT/*β*-catenin signaling triggered the activation of the CDX/HOX pathway, which in turn conferred a hematogenic posterior mesoderm phenotype to differentiating human iPSCs [57].

## 2. HOX Genes and Cancer

In recent years, it has been shown that HOX genes are not only responsible for proper embryonic development but they are also associated with cancer development [58]. In the next section of this review, we focus on the role of HOX genes in cancer development, particularly colorectal cancer (CRC) and acute myeloid leukemia (AML).

### 2.1. HOX Genes in CRC

Aberrant expression of HOX genes is seen in CRC [49, 50, 58, 59]. We previously reported that *HOXA4*, *HOXA9*, and *HOXD10* are expressed in normal colonic SCs and dysregulation of HOX genes leads to aberrant SC differentiation, contributing to CRC development and growth. Furthermore, we showed that *HOXA4* and *HOXA9* genes promote self-renewal and proliferation of colonic SCs, contributing to CRC development [49, 50].

**Figure 4:**
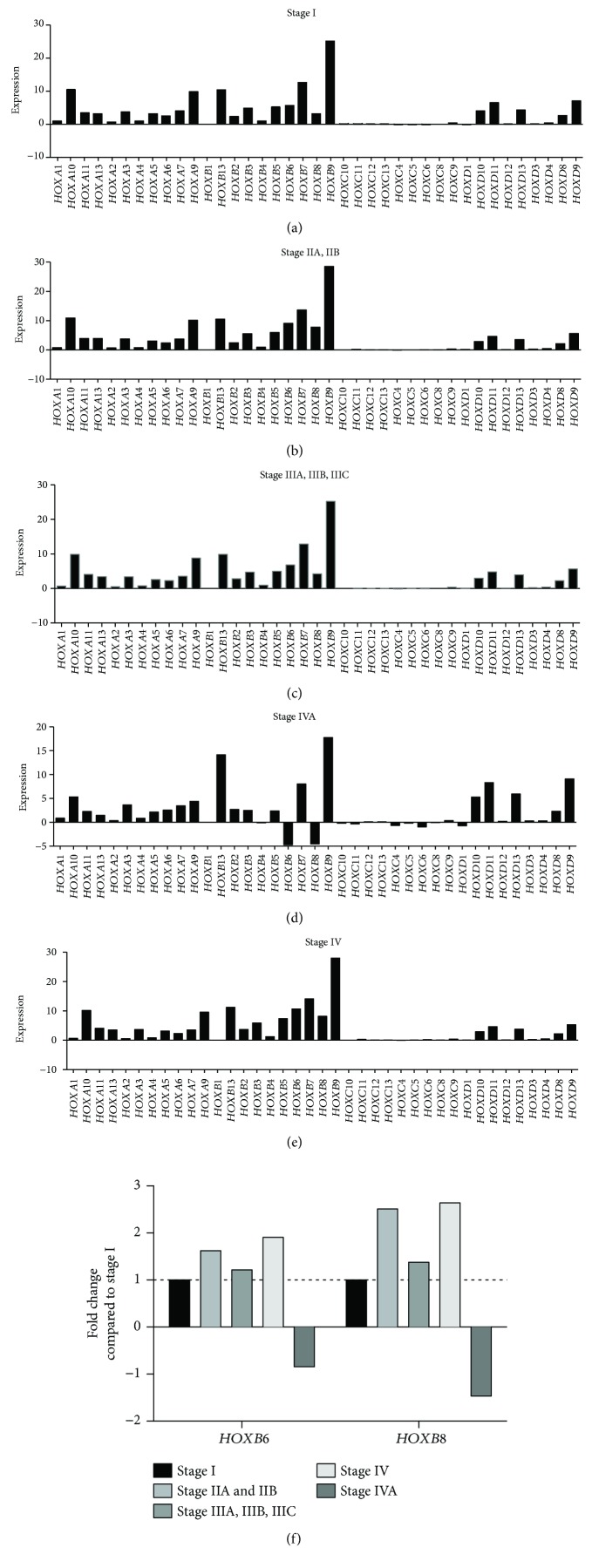
HOX gene expression during different stages of CRC. RNA sequencing data for CRC patients obtained from The Cancer Genome Atlas (TCGA) for HOX gene expression (normalized FPKM) and analyzed based on different stages of CRC. We studied (a) 55 cases for stage I, (b) 102 cases for stage IIA and IIB combined, (c) 66 cases reporting stage IIIA, IIIB, and IIIC, (d) 39 samples for stage IV, and (e) 4 cases for stage IVA. (f) Fold changes in the expression of *HOXB6* and *HOXB8* are shown for stages II, III, IV, and IVA compared to stage I.

In this review, we also evaluate the role of HOX genes during CRC development using The Cancer Genome Atlas (TCGA) database. Tumor samples were collected from newly diagnosed patients (i) with colon or rectum adenocarcinoma (ii) undergoing surgical resection, and (iii) having received no prior treatment for their disease, including no chemotherapy and no radiotherapy. All cases were collected regardless of surgical stage or histologic grade. Cases were staged according to the American Joint Committee on Cancer (AJCC) staging system. Each frozen tumor specimen had a companion normal tissue specimen which could be blood/blood components, adjacent normal tissue taken from greater than 2 cm from the tumor, or previously extracted germline DNA from the blood [60]. Our analysis showed that *HOXB9* was the most upregulated gene at all stages (Figure 4). *HOXB6* and *HOXB8* expression increased from stages I to IV but dramatically decreased at stage IVA. Interestingly, the expression of *HOXB6* and *HOXB8* was increased during stages I and II and decreased at stage III but again increased at stage IV (Figure 4(f)).

**Figure 5:**
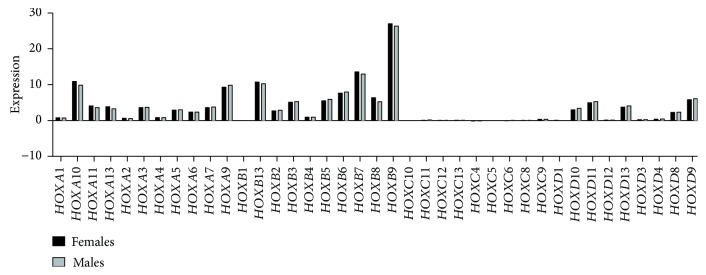
Gender-based HOX gene expression in CRC. The Cancer Genome Atlas (TCGA) was used to analyze gender-based differences in HOX gene expression in CRC patients. Normalized FPKM expression of HOX genes is plotted for male versus female CRC patients.

HOXD family gene expression increased at stage IVA compared to all other stages of CRC (Figure 4). Our analysis showed that there is no difference in HOX gene expression based on gender (Figure 5). When we compared TCGA datasets for HOX gene expression in CRC to overall survival, increased *HOXB13* was found to be associated with decreased survival (data not shown). It was reported earlier that missense germline *HOXB13* mutations, most commonly in G84E (HOXB13 p. Gly84Glu), are associated with early-onset prostate cancer and possibly associated with breast cancer and colorectal cancer [61–63]. Unlike *HOXB13*, increased *HOXB8* expression was associated with increased survival. We observed similar trends in expression of other HOX genes, whereby changes in the level of expression was correlated with improved survival and greater in tumor than tumor-free CRC cases (discussed below).

**Figure 6:**
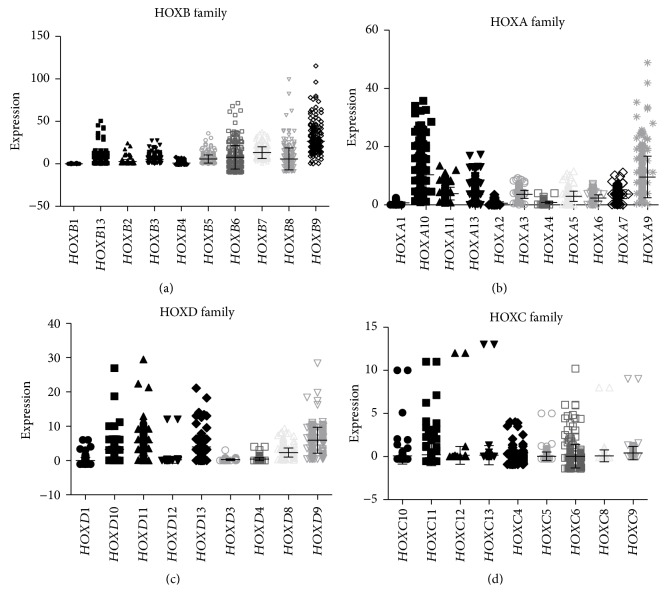
HOX family gene expression in CRC. (a) HOXB family gene expression, (b) HOXA family genes, (c) HOXD family genes, and (d) HOXC family genes were analyzed using TCGA RNAseq for *n* = 273 patient samples. *y*-axis denotes normalized FPKM values for HOX gene expression.

Next, we analyzed HOX expression based on their family gene clusters. Among all HOX families, the HOXB family showed the highest expression in CRC. In the HOXB family, *HOXB9*, *HOXB8*, *HOXB6*, *HOXB13*, *HOXB*, and *HOXB7* were all overexpressed in CRC (Figure 6(a)**)**. The HOXA family showed the next highest expression. In the HOXA family, *HOXA9* and *HOXA10* showed higher expression than the remaining HOXA family genes (Figure 6(b)). Among HOXD family members, *HOXD10*, *HOXD11*, *HOXD13*, and *HOXD9* showed increased expression compared to the others (Figure 6(c)). The HOXC family of genes showed the least expression compared to HOXA, HOXB, and HOXD (Figure 6(d)).

**Figure 7:**
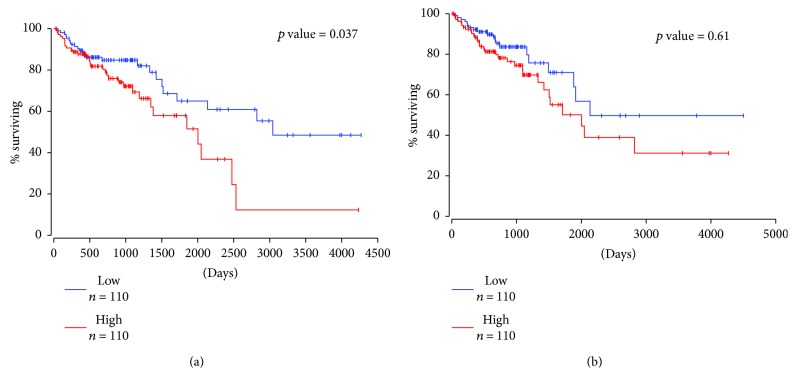
Overall survival analysis for *HOXA4* and *HOXD10* in colorectal cancer. Kaplan-Meier survival analysis of the 220 colorectal cancer patients using TCGA dataset. (a) *HOXD10* and (b) *HOXA4* survival analysis was performed for CRC patients with a cutoff value of 25th percentile. Credits: http://www.oncolnc.org.

**Figure 8:**
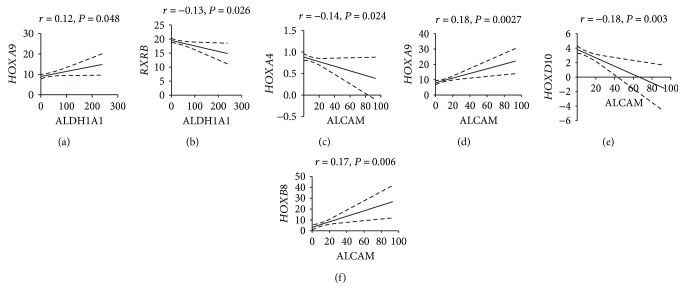
Correlation analysis of HOX genes and retinoid receptors with stem cell markers in colorectal cancer. (a) The expression of *HOXA9* and *ALDH1A1* (SC marker) in colorectal cancer (CRC) patients is correlated by Pearson correlation. A positive significant correlation was observed between *HOXA9* and *ALDH1A1* (*r* = 0.12, *P* = 0.048). (b) The expression of retinoid receptor *RXRB* and *ALDH1A1* in CRC patients is correlated by Pearson correlation. A negative significant correlation was observed between *RXRB* and *ALDH1A1* (*r* = −0.13, *P* = 0.026). (c) The expression of *HOXA4* and *ALCAM* (CD166, SC marker) in CRC patients correlated by Pearson correlation. A negative significant correlation was observed between *HOXA4* and *ALCAM* (*r* = −0.14, *P* = 0.024). (d) The expression of *HOXA9* and *ALCAM* in CRC patients correlated by Pearson correlation. A positive significant correlation was observed between *HOXA9* and *ALCAM* (*r* = 0.18, *P* = 0.0027). (e) The expression of *HOXD10* and *ALCAM* in CRC patients correlated by Pearson correlation. A negative significant correlation was observed between *HOXD10* and *ALCAM* (*r* = −0.18, *P* = 0.003). (f) The expression of *HOXB8* and *ALCAM* in CRC patients correlated by Pearson correlation. A positive significant correlation was observed between *HOXB8* and *ALCAM* (*r* = −0.17, *P* = 0.006).

We further assessed HOX genes as a function of overall patient survival. Because we previously found that *HOXA4* and *HOXD10* are expressed in crypt SCs, we measured *HOXA4* and *HOXD10* levels in CRC cases (*n* = 220) based on high versus low expression (cutoff at 25th percentile). *HOXA4* and *HOXD10* high-expressing cases (*n* = 110) of CRC patients showed overall low survival rates. Increased *HOXD10* expression was found to be significantly associated with poor overall survival in CRC (Figure 7(a)). *HOXD10* high expressers showed only a 15% survival rate versus 55% survival rate of *HOXD10* low expressers (Figure 7(a)). *HOXA4* high expressers showed about a 30% survival rate as compared to a 50% survival rate of *HOXA4* low expressers (Figure 7(b)). We also analyzed the association of *HOXA9* with the SC marker *ALDH1A1* in CRC patients using TCGA database. A significant positive correlation was observed between *HOXA9* and *ALDH1A1* in CRC (*r* = 0.12, *P* = 0.048) (Figure 8(a)). Retinoid receptors, *RXRB*, showed negative significant correlation with *ALDH1A1* (*r* = −0.13, *P* = 0.026) (Figure 8(b)). Another stem cell marker, *ALCAM* (also known as CD166), was correlated with HOX gene expression in CRC. Expression of *HOXA4* negatively correlated with *ALCAM* in CRC patients (*r* = −0.14, *P* = 0.024) (Figure 8(c)). *HOXA9* was positively correlated with *ALCAM* (*r* = 0.18, *P* = 0.0027) (Figure 8(d)). *HOXD10* showed significant negative correlation with *ALCAM* (*r* = −0.18, *P* = 0.003) (Figure 8(e)) whereas *HOXB8* showed a significant positive correlation with *ALCAM* (*r* = 017, *P* = 0.006) (Figure 8(f)).

Other published reports on the involvement of HOX genes in CRCs were reviewed [49]. The expression of *HOXD8* is downregulated in clinical CRCs compared to normal colon tissues, and the stable expression of *HOXD8* in CRC cells significantly reduced cell proliferation, anchorage independent-growth, and invasion. Apoptotic inhibitor genes such as STK38 and MYC were found to be negatively associated with *HOXD8* in analyses using The Cancer Genome Atlas (TCGA). Mansour and Senga demonstrated the ability of *HOXD8* to activate caspases 6 and 7 and cleave PARP, thus enhancing apoptosis of CRC cells [64]. A study by Chen et al. showed that *HOXD3* is upregulated in human RKO colon cancer cells. The inhibition of *HOXD3* by shRNA in RKO cells significantly decreased proliferation and colony formation and increased apoptosis of RKO colon cancer cells. *HOXD3*-inhibited cells were arrested in the G2 phase of the cell cycle [65]. Among HOXD clusters, induction of the expression of *HOXD8*, *HOXD9*, *HOXD10*, or *HOXD12* induces growth arrest and neuronal differentiation with downregulation of cell cycle-promoting genes and upregulation of differentiation genes. Other HOXD genes such as *HOXD1*, *HOXD3*, *HOXD4*, *HOXD11*, and *HOXD13* had no effects or only partial effects on neuroblastoma cell proliferation or differentiation [66]. These findings suggest that HOXD genes have distinct functions in the induction of cancer cell differentiation. Other HOX genes, such as *HOXB6*, *HOXB8*, *HOXC8*, *HOXC9*, and CDX1 were also found to be dysregulated in human CRC development [67]. Furthermore, the HOXA family gene was abundantly expressed in colonic adenocarcinoma cells [68]. Overall, these findings suggest that HOX genes play key regulatory roles during maintenance of normal colon SC differentiation and that aberrant expression is associated with CRC development.

#### 2.1.1. CDX1/CDX2 Genes in CRC

Another set of Hox genes, the Cdx genes (also classified as ParaHox genes or caudal-related homeobox genes), is expressed in a wide variety of organisms [69]. Three Cdx genes, *Cdx1*, *Cdx2*, and *Cdx4*, exist in mouse and humans (CDX1, CDX2 and CDX4) and regulate anterior-posterior patterning [70, 71]. The CDX genes are not located in a homeobox cluster, *CDX1* is located on human chromosome 5q31–33, *CDX2* gene is on chromosome 13q12, and *CDX3* is on Xq13.2. In mice, *Cdx1* and *Cdx2* are important for gastrointestinal tract development. *CDX1* and *CDX2* are also actively expressed in adult intestinal epithelium and are involved in the regulation of enterocyte proliferation and differentiation as well as WNT-mediated beta-catenin signaling [70, 72, 73]. Moreover, reduced expression of *CDX2* appears to contribute to the development of intestinal neoplasia and is a prognostic biomarker for stage II and stage III colon cancer by identifying high-risk patients who might benefit from adjuvant chemotherapy [74–76].

#### 2.1.2. HOX Genes in AML

**Figure 9:**
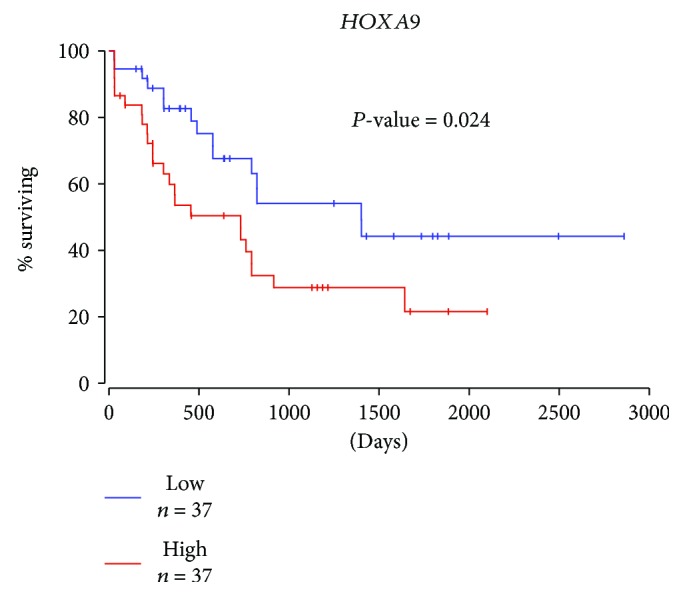
Overall survival analysis for *HOXA9* in acute myeloid leukemia. Kaplan-Meier survival analysis of *HOXA9* in acute myeloid leukemia (AML) using TCGA dataset. Survival analysis was performed for AML patients (total *n* = 74) with a cutoff value of 25th percentile. Credits: http://www.oncolnc.org.

Studies of AML patients show that many cases (~35%) have mutations in type III receptor tyrosine kinase FLT3 and that *HOXB2* and *HOXB3* are increased in AML patients with FTL3-ITD (internal tandem duplication) mutation. The overexpression of *Hoxb2* and *Hoxb3* in mouse pro-B cells resulted in decreased FLT3-ITD-dependent cell proliferation, reduced colony formation, and increased apoptosis, suggesting that *HOXB2* and *HOXB3* are regulators of FLT3-ITD-driven AML [77]. Several studies also showed that HOX genes promote AML development by forming chimeric fusions with other genes. Fusion of the nucleoprotein NUP98 with HOXA9 via chromosome translocation t(7;11) (p15s;p15) causes development of AML [78]. Mice overexpressing *Hoxa9* and *Meis1a* induced growth factor-dependent AML in less than 3 months. However, the overexpression of *Hoxa9*, *Mesi1a*, or *Pbx1b* alone, or in combination with *Hoxa9* and *Pbx1b*, failed to transform these cells acutely within 6 months posttranslation [79]. *NUP98-HOXA9* fusion genes induced long-term proliferation and blocked differentiation of human CD34+ HSCs [80]. Recent data showed that mixed lineage leukemia (MLL) is crucial for *NUP98-HOXA9* leukemia initiation [81]. We analyzed the TCGA dataset and did overall survival analysis for *HOXA9* in AML patients (*n* = 74) and found that *HOXA9* high expressers had a 20% survival rate compared to *HOXA9* low expressers which had a 50% survival rate (Figure 9).

### 2.2. Cancer Stem Cells

Cancer stem cells (CSCs) are multipotent and have the ability to undergo both self-renewal and differentiation. We and others have shown that CSCs are the root cause of cancer development [46, 47]. These CSCs are resistant to chemotherapy and radiation. Previously, we identified *HOXA4*, *HOXA9*, and *HOXD10* signatures for normal colonic SCs and that these HOX genes are upregulated during CRC development. Indeed, *HOXA4* and *HOXA9* were found to have roles in self-renewal and proliferation of colonic SCs that contribute to CRC development [49, 50]. Moreover, *HOXA9* is known to have a pivotal role in HSC self-renewal and that the upregulation of *HOXA9* leads to AML [82]. Another report showed that *miR-375* inhibited the proliferation of CSCs and tamoxifen resistance by targeting *HOXB3* in human ER-positive breast cancers [83].

### 2.3. HOX Genes as Biomarkers

HOX genes have been used as markers to distinguish stromal populations from different tissue sources. The results show that the stromal populations have distinct HOX signatures with different growth and differentiation abilities although they are all immuno-phenotypically similar. These stromal cell populations express different HOX genes and their level of expression varies. Overall, these results indicate that HOX gene profiles can be used to provide positional, embryological, and hierarchical identity of human stromal stem cells [84].

## 3. Mechanisms Involved in HOX Gene Dysregulation in Cancer

### 3.1. Aberrant Self-Renewal and Proliferation

We have shown that *HOXA4* and *HOXA9* are upregulated in CRC SCs [49, 50] and that siRNA knockdown of *HOXA4* and *HOXA9* reduces proliferation and sphere-formation ability of CRC SCs. *HOXA4* and *HOXA9* knockdown also changed the expression of SC markers, such as *ALDH1*, *ALCAM*, (CD166) and *LGR5*. Treatment of CRC cells with the differentiating agent all-*trans*-retinoic acid (ATRA) decreased *HOXA4*, *HOXA9*, and *HOXD10* expression in parallel with decreases in SC levels. Overall, our study demonstrated a role for HOX genes in self-renewal and proliferation of CRC SCs. Thus, strategies designed to modulate HOX expression may provide a means to target malignant SCs and to develop more effective therapies for CRC [50].

Notably, the self-renewal ability of *HOXB4* is dependent upon a proline-rich sequence near the N terminus, which is also unique and highly conserved among the other HOX proteins. Deletion of this domain significantly enhanced the oncogenicity of *Hoxb4*, promoting features of acute leukemia in mice. Insertion of this domain into *Hoxa9* impaired oncogenic potential for leukemia. Overall, this study showed that such proline-rich stretches in HOX genes attenuate the potential of SCs to acquire oncogenic properties [85].

## 4. HOX Genes and Related Therapeutics

### 4.1. HOTAIR Long Noncoding RNA (lncRNA)

HOTAIR is a 2.2 kilobase *trans*-acting lncRNA residing in the HOXC loci that function to repress transcription of 40 kilobases of the HOXD locus. HOTAIR has been shown to interact with polycomb repressive complex 2 (PRC2). Its interaction with PRC2 is required for PRC2 occupancy and histone H2 lysine-27 trimethylation of the HOXD locus [86]. HOTAIR has been proposed as a biomarker in cervical cancer [87], nasopharyngeal carcinoma [88], and gallbladder cancer [89]. Indeed, meta-analysis involving 748 patients from 8 studies showed HOTAIR is a molecular marker for lymph node metastasis. The results indicated a significant difference in the incidence of lymph node metastasis between high and low HOTAIR expression groups [90]. HOTAIR lncRNA plays a crucial role in epithelial-mesenchymal transitions and is required for the maintenance of colon and breast cancer stem cell stemness [91]. Overall, HOTAIR lncRNA has potential as a therapeutic target in several cancer types. A recent study showed that expression of HOTAIR increased in CRC cells and cell lines and HOTAIR knockdown promoted apoptosis and inhibited proliferation, migration, and invasion *in vitro* and *in vivo*. Furthermore, HOTAIR modulated CRC progression by competitively binding *miR-197* [92].

### 4.2. PBX/HOX Dimer

One of the mechanisms that regulates HOX transcriptional expression is through binding with PBX proteins. Both HOX and PBX proteins are known to play critical roles in carcinogenesis, which makes it an attractive therapeutic target for cancers. One study showed that *PBX3* is a potential pathologic cofactor of *HOXA9* involved in cytogenetically abnormal acute myeloid leukemia (CA-AML), particularly MLL-rearranged AML. The depletion of *PBX3* expression by shRNA significantly inhibited MLL-fusion-mediated cell transformation, whereas coexpression of *PBX3* with *HOXA9* promoted cell transformation *in vitro* and leukemogenesis *in vivo*. A small peptide, known as HXR9, that disrupts the interaction between HOX and PBX proteins was found to be effective in killing leukemic cells that were overexpressing *HOX/PBX3* genes, which suggests a potential therapeutic strategy for CA-AML patients [93]. HXR9 has anticancer effects in other tumor types, such as breast [94], mesothelioma [95], ovarian [96], meningioma [97], prostate [98], and non-small cell lung [99]. Additionally, the disruption of HOXB7/PBX2 proteins by HXR9 is a potential therapeutic target in malignant melanoma [100]. A recent study also showed that the expression of *HOXA5*, *HOXB2*, *HOXB4*, *HOXB9*, and *HOXC9* (but not *HOXA9*) in primary AML cases is significantly correlated with survival. HXR9 treatment is cytotoxic to AML-derived cell lines and primary AML cells from patients. And it was shown that cell death is independent of apoptosis. Rather, it involves necroptosis (a regulated form of necrosis) [101]. This study suggests that HXR9 treatment for cancers should be seriously explored in future studies. In addition to the upregulation of *HOXA4*, *HOXA9*, and *HOXD10* in CRC [49, 50, 58], the *PBX* genes are also overexpressed in CRC, which correlates with invasive potential *in vitro* and lymph node invasion, distant metastasis, advanced TNM stage, and poor overall survival of patients [102]. These reports suggest that HXR9 treatment in CRCs might be therapeutically useful to target HOX/PBX proteins in CRCs.

## 5. Conclusion

The above findings suggest that (i) HOX genes play diverse roles in normal SC functions and properties, from self-renewal to multilineage differentiation, and (ii) the dysregulation of HOX genes contributes to cancer development through aberrant self-renewal and differentiation of SCs. Thus, understanding the molecular mechanisms for how HOX genes control SC self-renewal and differentiation will ultimately help us understand how SC populations are maintained in normal, disease-free states and how the dysregulation of HOX genes leads to abnormal SC self-renewal and differentiation that drive cancer development. Ultimately, understanding the mechanisms by which HOX genes are regulated in SC might help to find ways to manipulate SC fate resulting in the development of novel, more effective SC-targeted treatments for cancer.
